# Patient satisfaction with two different models of cancer genetic services in south-east Scotland

**DOI:** 10.1038/sj.bjc.6601562

**Published:** 2004-02-03

**Authors:** S Holloway, M Porteous, R Cetnarskyj, E Anderson, R Rush, A Fry, D Gorman, M Steel, H Campbell

**Affiliations:** 1 Department of Clinical Genetics, Molecular Medicine Centre, Western General Hospital, Crewe Road South, Edinburgh EH4 2XU, UK; 2 Department of Public Health Sciences, University of Edinburgh Medical School, Teviot Place, Edinburgh EH8 9AG, UK; 3 Edinburgh Breast Unit, Western General Hospital, Crewe Road South, Edinburgh EH4 2XU, UK; 4 SE Scotland Breast Screening Service, Ardmillan House, Ardmillan Terrace, Edinburgh EH11 2SL, UK; 5 Cancer Research UK, Edinburgh Oncology Unit, Western General Hospital, Crewe Road South, Edinburgh EH4 2XR, UK; 6 Lothian NHS Board, Deaconess House, 148 Pleasance, Edinburgh EH8 9RS, UK; 7 School of Biology, University of St Andrews, St Andrews, Fife KY16 9TS, UK

**Keywords:** breast cancer, cost, expectations, genetic counselling, satisfaction

## Abstract

There is a need to integrate primary- and secondary-care cancer genetic services, but the most appropriate model of service delivery remains unclear. This study reports patients’ expectations of breast cancer genetic services and a comparison of their satisfaction with two service models. In the first model, risk assessment was carried out using mailed family history data. Women estimated as being at high/moderate risk were offered an appointment at the familial breast cancer clinic, and those at low risk were sent a letter of reassurance. In the second model, all women were seen by a genetic nurse specialist, who assessed risk, referred high/moderate-risk women to the above clinic and discharged those at low risk. Over 60% of all women in the study regarded access to breast screening by mammogram and regular check-ups as very important. This underlines the demand for a multidisciplinary service providing both clinical genetic and surgical services. Satisfaction was high with both models of service, although significantly lower among women not at increased cancer risk and thus not offered a clinical check-up and mammography. Increased cancer worry was associated with a greater expressed need for information and for reassurance through follow-up clinical checks and mammography. Better targeting of counselling to the expressed concerns and needs of these women is required to improve the service offered. GPs and patients expressed no clear preference for any specific service location or staffing configuration. The novel community service was less expensive in terms of both staff and patient costs. The potential to decrease health staff/patient contact time and to employ nurse practitioners with both clinical genetic and oncology training should be explored further. The rapidly rising demand for these services suggests that the evaluation of further new models needs to continue to be given priority to guide the development of cancer genetic services.

Media attention to scientific developments in cancer genetics and increased public awareness of the potential importance of a family history of cancer has resulted in a greatly increased demand for cancer genetic services. These services aim to identify individuals who have inherited a significantly increased risk of cancer in order to counsel them about their risks and to offer appropriate risk management to reduce morbidity and mortality. Genetic counselling for patients with a family history of cancer has been shown to result in a more accurate perception of risk (Evans *et al*, 1994) without an increase in anxiety (Hopwood *et al*, 1998). A survey of 22 regional cancer genetic services in the UK in 1998 reported that the predominant users of these services were women with a family history of breast cancer (Wonderling *et al*, 2001). Internationally, there is a lack of consensus about how best to deliver cancer genetic services (Steel *et al*, 1999), and an urgent need for empirical evidence to inform service development within the existing healthcare budgets.

A model of cancer genetic services has been proposed (Campbell *et al*, 1995, 2003; Fry *et al*, 2003), whereby genetic nurse specialists could offer clinics within GP locality areas to carry out risk assessment, provide counselling for those whose risk was not significantly increased and mediate referral of those at higher risk to the specialist service. It was hoped that this would provide improved support to primary care and better services for those not at increased risk, while encouraging more cost-effective use of specialist resources for those at increased risk of developing breast cancer. We have previously reported that the establishment of community-based clinics leads to substantially higher rates of annual referral, less evidence of inequity of access due to deprivation and improved referral practices (Campbell *et al*, 2003), but not to improved patient outcomes (Fry *et al*, 2003). In this study, we report women's expectations of cancer genetic services and the results of a trial assessing women's satisfaction with this new model of service.

## MATERIALS AND METHODS

### Participants

Ethical approval for the study was obtained from the local ethics committee. An invitation to take part in the trial was sent to all general practices in Lothian (*n*=125), south-west Fife (*n*=54) and Borders (*n*=24) Health Boards in south-east Scotland. In all, 179 practices (84%) agreed to take part, 23 (11%) declined and 10 (5%) did not reply. This meant that 725 of the 828 (88%) GPs in practice across these three Health Boards agreed to refer patients into the trial. Practices were randomly assigned to either arm of the trial using a minimisation technique (Pocock, 1983, pp 84–86) to ensure that the two groups were balanced for: size of practice; historical referral rate; and social deprivation index.

During the period March 1998 to November 1999, any woman referred from participating GP practices to the regional clinical genetics department for breast cancer genetic risk counselling was invited to take part in the trial. To be eligible for the trial, women had to live in the region, be able to give informed consent and to complete a baseline questionnaire. Women who were symptomatic or had been diagnosed with breast and/or ovarian cancer were excluded from the trial as were those who had previously consulted another clinic about their family history of cancer. Those who were ineligible to participate were offered the standard regional service.

### Procedures

The service offered to women who returned a consent form and a completed baseline questionnaire was dependent on the arm of the trial to which their GP practice had been randomised. Details of the trial procedures have been described in detail (Campbell *et al*, 2003; Fry *et al*, 2003), but briefly the trial groups were:

#### Standard (regional) service

Women were sent a family history form to complete. The family history form requested information about first-, second- and third-degree relatives. If the family history form was not returned, a letter was sent to the woman and to her GP to explain that no consultation was possible without this information. The genetic nurse specialist drew a pedigree from the information on the family history form, then assigned categorical risk assessments together with a genetics consultant using the criteria published by the Cancer Research Campaign (Cancer Research Campaign, 1997). If necessary, further information and/or confirmation of relatives’ diagnoses were obtained from the cancer registry. When a woman was assessed as not being at a significantly increased risk (i.e. ‘low’ risk), she and her GP were sent a letter to explain this. An appointment at the familial breast cancer clinic was offered to women assessed as being at ‘moderate’ or ‘high risk’, and those for whom an adequate risk assessment could not be made from the information available. The clinic consultation offered more detailed discussion with a genetics consultant about risk status and with a specialist breast surgeon about options for risk management. Clinical breast examination and mammography (where appropriate) were carried out at this visit. After this appointment, the patient's GP was sent a letter to summarise the issues discussed. All women were asked to complete a postal follow-up questionnaire 4 weeks and 6 months later.

#### Novel (community-based) service

All women in this arm of the trial were sent an initial appointment for one of the community-based clinics (held in a GP practice near to where they lived), run by a genetic nurse specialist. At the clinic, the genetic nurse specialist ascertained the woman's family history of cancer and compiled a family tree. This information was compared to the criteria published by the Cancer Research Campaign (Cancer Research Campaign, 1997) to determine whether she was at a significantly increased risk. When an adequate risk assessment could not be made during the appointment, further information and/or confirmation of relatives’ diagnoses were obtained from the patient, medical records or the cancer registry before the patient was informed of their risk by letter. Women deemed not to be at a significantly increased risk (i.e. in the ‘low-risk’ category) were offered information and reassurance and were discharged from the clinic. These women and their GPs were sent a letter reaffirming their ‘low-risk’ status and summarising the issues discussed at the appointment. The women were asked to complete a postal questionnaire 4 weeks and 6 months later. Women found to be at increased risk (i.e. in the ‘moderate-risk’ or ‘high-risk’ categories) were offered an appointment at the regional centre with a consultant breast surgeon and a genetic nurse specialist. Prior to this consultation, they were sent a questionnaire asking for their opinions of the community clinic appointment and what further information or services they wished from the regional clinic. They were asked to complete a postal follow-up questionnaire 4 weeks and 6 months after their clinic appointments.

### Sociodemographic and objective breast cancer risk data

Women were asked to record their date of birth, marital status and educational level on the baseline questionnaire. Information was also requested on mode of referral, knowledge of breast cancer and its inheritance, psychological status and details of what services and information was sought from the consultation. Information about the category of breast cancer risk to which each woman had been assigned was derived from the clinical records.

### Data relating to the consultation

#### Clinic data

The details of all clinic consultations were recorded. These included duration of consultation, level of risk stated, matters discussed, time spent in various clinic activities and outcome of the consultation. Matters discussed at the consultation were classified under five headings (family history and genetics; examination and screening; healthy lifestyles; other matters related to breast cancer and other matters unrelated to breast cancer).

#### Satisfaction with services received

At the 4-week and 6-month follow-up, satisfaction with the consultations was measured in several ways. To assess general satisfaction, women were asked to assess a number of items from the Medical Interview Satisfaction Scale (MISS) (Wolf *et al*, 1978; used with permission from the author). We used 17 of the 26 original questions in the three subscales. The psychometric properties of this scale have been investigated in surveys in general practice with a conclusion that the MISS represents ‘a valid and reliable instrument for the assessment of patient satisfaction with individual consultations in British general practice’ (Meakin and Weinmann, 2002). Satisfaction with three aspects of a consultation were measured:

- *The affective aspect (A)*: The extent to which the respondent feels the medical professional (MP) listens, understands and is interested.
- *The behavioural aspect (B)*: The respondent's evaluation of the MP's competence in the consultation.
- *The cognitive aspect (C)*: Satisfaction with the amount and quality of information provided by the MP.

Each item on the scale was rated on a five-point scale of agreement from strongly agree (5) to strongly disagree (1). The summed scores were divided by the number of items answered by the subject to give mean scores for each aspect of the consultation and an overall mean score. An evaluation of the subscales within UK general practice has shown that they represent fairly discrete but overlapping aspects of satisfaction (Meakin and Weinmann, 2002).

We also investigated patients’ assessment of the helpfulness of the specific information given and services offered at the consultation. We asked what additional information/services women would have wished to receive and what further action they had taken since their attendance at the clinic. We also asked about their preferences with respect to the clinic location and staffing.

#### Other measures

Psychological distress and cancer worry were measured at baseline by the General Health Questionnaire (GHQ 30) (Goldberg and Williams, 1988) and the Cancer Worry Scale (Watson *et al*, 1998) as described by Fry *et al* (2003).

#### Relative cost of operating novel and standard service clinics

(a) *Estimate of staff costs*:

We estimated staff time taken for various aspects of the consultation (such as pedigree drawing, risk assessment and counselling) and travel time to clinics by asking the staff to complete a standard form recording these details. We also recorded details of women's attendance and nonattendance at clinics. Relative costs were based on a medical salary being two times that of a clinical genetic nurse specialist (consultant or associate specialist annual salary of £50 000 and clinical genetic nurse specialist salary of £25 ,000). The estimates also assumed that secretarial and administrative staff costs for the novel and standard service models were approximately equal, with the support for additional clinics in the novel service being offset by that for obtaining family history forms from all patients and the higher nonattendance rate in the standard service. A further assumption was that the efficiency of staff use within the clinics could be made approximately equal in the two service models by appropriate management of clinic sizes and appointments.

(b) *Estimate of patient time and financial costs:*

Patients were asked to complete a short questionnaire after clinic appointments asking them to give details of how they reached the clinic, their travel time and costs, details of any other costs (such as child care) and any loss of earnings and details of normal activities interrupted by the appointment.

## RESULTS

### Participants

Figure 1

**Figure 1:**
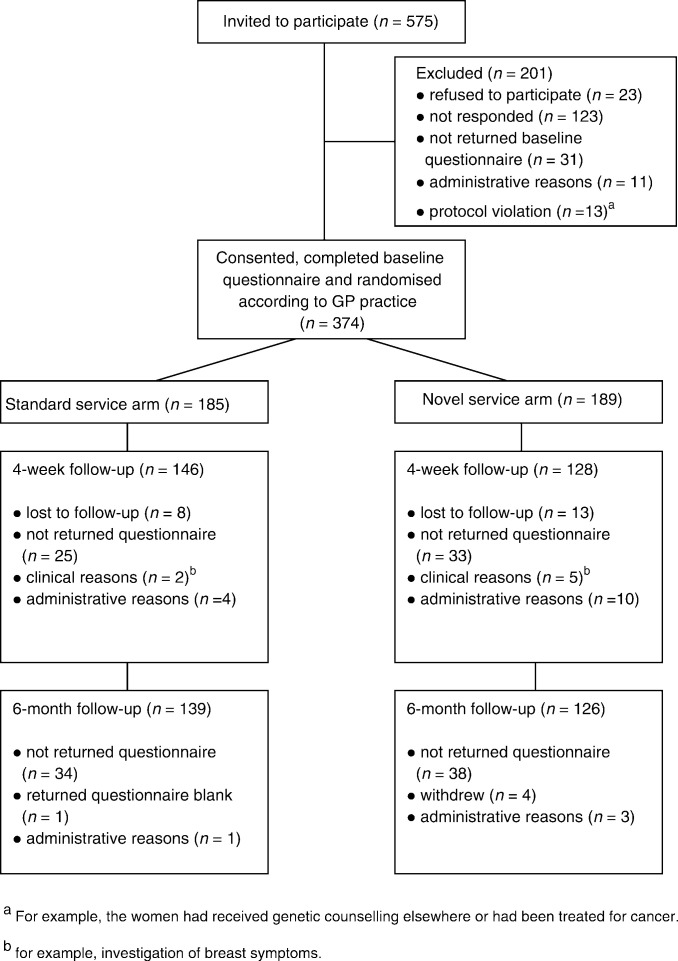
Progress of participants through the trial.

shows the progress of participants through each arm of the trial with respect to the completion of questionnaires described in this report.

#### Baseline

Over the study period, 374 women consented to take part in the trial and completed a baseline questionnaire. The age of the women ranged from 17.5–69.6 years with a mean (±s.d.) of 38.5±9.5 years. The characteristics of these women are described in a related publication (Fry *et al*, 2003). There were no differences in age, sociodemographic or educational factors between the two trial groups (Fry *et al*, 2003).

#### Follow-up

The completion rates for the follow-up questionnaires are presented in Figure 1. A total of 274 (73%) completed 4-week and 265 (71%) women completed 6-month follow-up questionnaires. There were no significant differences between the questionnaire completion rates in the two arms of the trial.

### Clinic consultations

Information was recorded on 379 clinic consultations. The genetics of breast cancer, the significance of the family history and the patient's own risk were discussed in almost all first consultations. In a large proportion (45–86%) of these consultations, there was also discussion of risks to children and other relatives and the possibility of finding a cancer predisposing gene. Mammography was discussed in almost all consultations. Screening for other cancers was much more likely to be discussed by the doctors seeing women who received the standard (regional) service. Breast self-examination and adoption of healthy lifestyles were more likely to be discussed by the nurse at the community clinic (novel service).

The length of time in minutes spent on each part of the consultation was not normally distributed in any of the consultation groups. The median total consultation time was 41 min for the community clinic appointment and 12 min for the regional follow-up appointment as part of the novel service; and 18 min for the regional appointment of the standard service. Women receiving the novel service spent more time at their initial community clinic appointment discussing each of the five areas of consultation (see above) than those receiving the standard service (*P*<0.01 to 0.001, Mann–Whitney test). Some of the increased time taken by the novel service was due to the fact that details of the family history had to be obtained at the community clinic appointment rather than by questionnaire as in the standard service.

#### Choice of clinic location and personnel

At the 4-week follow-up, 107 (96%) women were satisfied with the length of community clinic appointment and 89–93% of women with that at the regional clinic. In all, 69 (30%) women did not state any strong preference for the location of a familial breast cancer clinic run by specially trained staff. There was a tendency for women to prefer the location of the clinic they had attended. The most popular choices were for a community clinic (selected by 27 (52%) of low-risk women who had received the novel service) and a regional clinic (selected by 51 (42%) women who received the standard service). Among the women who had attended both community and regional clinics, 23 (38%) preferred a community clinic and 16 (27%) a regional clinic. Overall, 115 (50%) of the women expressed no strong preference on the grade and type of staff and 58 (25%) preferred a genetic nurse and a consultant breast surgeon.

### Expectations of the breast cancer family clinic

#### Information needs

In all, 294 (79%) women said that they would like as much information as possible about their family history of cancer, but a minority of 35 (9%) wanted general information only and 43 (12%) only wished to know if their family was at increased risk. Women in the first group had significantly (*P*<0.05) higher cancer worry scores than women in the other two groups combined (Mann–Whitney test).

Women were asked to rate how important it was for them to get information about various specific issues. Items of information regarded by over 70% of women as very important are given in Table 1

**Table 1:** Information and service requirements of patients

***Items of information regarded by over* 50% *of women as very important***
The significance of their family history
Their own risk of breast cancer
Anything they can do in everyday life to reduce their cancer risk
How to examine their own breasts
Symptoms of breast cancer to look for
The pros and cons of breast screening
Research to find new or better ways to prevent/detect breast cancer
Breast cancer and its treatment

*Services for which access was regarded as very important by over* 60% *of women*
Reassurance that they show no signs of cancer now
Breast screening by mammogram
Regular check-ups

. Women less than 40 years attached greater importance to getting information about their risk than did older women (*P*<0.01).

#### Access to specific services

Services for which access was regarded as very important by over 60% of women are given in Table 1. Women who placed great importance on the need for services to check their current cancer risk (those who rated the need for breast examination, check for current signs of cancer and mammography as very important) showed no difference in objective cancer risk or anxiety levels compared to those who did not. However, these women exhibited significantly greater cancer worry (*P*<0.01, Mann–Whitney test).

### Assessment of services received

#### Patient satisfaction with services: overall satisfaction

Table 2

**Table 2:** Median satisfaction subscale scores (with 25th and 75th percentiles) by trial group (modified MISS)

**Patient group**	**Affective (A) scale**	**Behavioural (B) scale**	**Cognitive (C) scale**
Novel service: all women	4.0 (3.6–4.3)	4.2 (3.9–4.6)	4.0 (4.0–4.6)
Standard service: all women	4.0 (3.7–4.4)	4.4 (4.0–4.6)	4.0 (4.0–4.8)

MISS=Medical Interview Satisfaction Scale.

summarises the median patient satisfaction scores by MISS subscale and trial group. There were no significant differences by trial group.

Table 3

**Table 3:** Numbers and percentages of women who agreed/strongly agreed^a^ with various statements concerning their appointments

**Statement**	**Aspect of consultation**^b^	**Novel service (community clinic) low-risk women**	**Novel service (community clinic) moderate/high-risk women**	**Novel service (regional clinic) moderate/high-risk women**	**Standard service (regional clinic) moderate/high-risk women**
(a) I was told about my risk of developing cancer in words that I could understand	C	31 (91.2%)	77 (98.7%)	54 (96.4%)	82 (95.3%)
(b) After the consultation I have a good idea of what changes in my health I should seek medical advice about.	C	20 (64.6%)	67 (85.9%)	47 (85.5%)	65 (82.3%)
(c) At the consultation I was told all I wanted to know about my family history of breast cancer	C	26 (74.3%)	71 (93.5%)	47 (84.0%)	81 (92.0%)
d) The person I saw was very good at explaining the reasons for any medical tests which may be necessary	C	22 (81.4%)	67 (95.7%)	48 (88.9%)	76 (93.8%)
e) I feel I understand pretty well the plan for helping me	C	14 (56.0%)	67 (94.4%)	52 (96.3%)	79 (93.0%)
(f) I was given a chance to say what was really on my mind	A	30 (88.2%)	64 (90.1%)	46 (86.7%)	74 (85.0%)
(g) I really felt I was understood	A	22 (64.7%)	63 (87.5%)	43 (82.7%)	69 (84.2%)
(h) After the consultation I felt much better about my problems	A	16 (53.3%)	46 (69.7%)	39 (78.0%)	59 (76.6%)
(i) I felt the person I saw really knew how upset I was about my family history	A	19 (63.3%)	42 (75.0%)	35 (79.5%)	41 (64.0%)
(j) I felt free to talk about private thoughts	A	23 (71.9%)	46 (70.8%)	37 (77.1%)	48 (64.0%)
(k) I felt accepted as a person	A	32 (91.4%)	64 (91.4%)	50 (92.6%)	74 (93.7%)
(l) I felt that my problems were not taken seriously	A	24 (75.0%)	63 (92.7%)	43 (89.6%)	79 (96.3%)
(m) All the problems I mentioned were looked into	B	21 (70.0%)	57 (86.4%)	38 (90.5%)	60 (86.9%)
(n) I felt the person I saw did not spend enough time with me	B	32 (94.1%)	74 (94.9%)	54 (96.5%)	79 (94.0%)
(o) I was satisfied with the advice I was given about the courses of action I could take.	B	19 (59.4%)	75 (97.4%)	49 (90.8%)	75 (88.2%)
(p) The person I saw seemed rushed during the consultation	B	33 (97.0%)	75 (96.2%)	54 (96.4%)	80 (94.1%)
(q) The person I saw gave me too much information too quickly	B	30 (85.7%)	69 (88.5%)	49 (87.5%)	82 (94.3%)

a or disagreed/strongly disagreed items l, n, p, q.

b A=affective aspect (doctor/nurse listens, understands and is interested); B=behavioural aspect (doctor/nurse competence); C=cognitive aspect (amount and quality of information provided by doctor/nurse).

details the views of patients on specific aspects of satisfaction with services at the 4-week follow-up. The items are listed together with the aspect of the consultation measured and the number and percentage of patients who agreed/strongly (dis)agreed with the statement. A single satisfaction score was constructed as the mean of scores of all 17 questions. Most women were satisfied with the consultations in both models of service, with responses heavily skewed towards the ‘satisfied’ responses. When we considered factors that may have influenced satisfaction, no statistically significant correlation (Spearman rank correlation) was found between overall score and cancer worry (Cancer Worry score), anxiety (GHQ score), age or deprivation score. There was no significant difference between satisfaction in different educational groups (ANOVA). However, women assessed as ‘low’ risk were less satisfied with the services they received (*P*<0.05, *t*-test) than those assessed at ‘moderate’ or ‘high’ risk, as defined in this study.

#### Patient satisfaction with services: satisfaction subscales (see Table 3)

The scores for the affective, behavioural and cognitive subscales in the MISS were not significantly correlated (Spearman rank correlation) with cancer worry (Cancer Worry score), anxiety (GHQ score) or age. There was no significant difference between educational groups in any of these scores (Kruskal–Wallis test). However, women at ‘low’ risk of breast cancer gave significantly lower mean scores for the affective (listening, understanding and interest of health staff) and cognitive (amount and quality of information given), but not the behavioural (competence of health staff) components of the satisfaction questionnaire (*P*<0.05, Mann–Whitney test).

#### Additional services requested by women

A greater proportion of women who received the novel community service stated at the 4-week and 6-month follow-up that they would have liked additional services (Table 4

**Table 4:** Numbers (percentages) of women who stated that they would have liked additional services (not offered to them at the clinic consultation)

**Patient group**	**4-week follow-up**	**6-month follow-up**
Novel service: all women	26/115 (23%)	17/114 (15%)
Novel service; low-risk women	19/53 (36%)	11/50 (22%)
Novel service; high/moderate-risk women	7/62 (11%)	6/64 (9%)
		
Standard service: all women	23/124 (19%)	14/117 (12%)
Standard service; low-risk women	5/12 (42%)	1/8 (13%)
Standard service; high/moderate-risk women	18/112 (16%)	13/109 (12%)

). However, this is confounded by the higher proportion of women scored as low risk in the novel service trial group. Overall, in both trial groups 37% (24 of the 65) low-risk women wished access to other services. Low-risk women receiving the novel service noted mammography, breast examination, regular check-ups and screening for other cancers most commonly. Eight (19%) of these women at 4 weeks and seven (17%) women at 6 months wanted access to mammography. High- and moderate-risk women receiving the standard service most commonly noted screening for other cancers and genetic testing. At the 4-week follow-up, women who wanted further appointments to check their breast cancer status had higher cancer worry scores than other women (*P*<0.05, Mann–Whitney test).

#### Further action since attending clinic(s)

Table 5

**Table 5:** Numbers (percentages) of women who intended to and had sought further advice

**Patient group**	**Intending to seek further advice**	**Had sought advice**
Novel service: all women	14/ 99 (14%)	7/99 (7%)
Novel service; low-risk women (*n*=42)	5 (12%)	4 (10%)
Novel service; high/moderate-risk women (*n*=57)	9 (16%)	3 (5%)
		
*Standard service: all women*	28/111 (25%)	11/111 (10%)
Standard service; low-risk women (*n*=8)	0	0
Standard service; high/moderate-risk women (*n*=103)	28 (27%)	11 (11%)

shows the number (%) of women (who completed both the 4-week and 6-month questionnaires) who stated that they intended to seek and had sought further advice about their family history of cancer after their clinic appointments. Overall, 42 (20%) stated that they intended to seek further advice and 18 (9%) actually sought further advice within 6 months. Most women simply wanted to keep up to date with new research or to find out about matters that they had not asked about at the clinic visits. A higher proportion of women receiving the standard service than the novel service (*χ*^2^ test, *P*<0.05) and of women at moderate or high risk than low risk (*χ*^2^ test, *P*<0.05) stated that they intended seeking such advice.

### Women receiving standard service who did not attend a clinic but received a letter only

Women in the standard service group who were assessed at low risk were not offered a clinic appointment but were sent a letter explaining that they were not at increased risk of breast cancer. Some 22 (73%) of these women returned a questionnaire at 4-week and at 6-month follow-up. Although 15 (68%) found the information in the letter quite or very helpful, seven (32%) found it only a little helpful or not at all helpful.

Six (27%) and eight (36%) women, respectively, noted that there were other items about which they would have liked information at the 4-week and 6-month follow-up. Seven (33%) and eight (38%) women stated that they would have preferred a clinic appointment to a letter at the 4-week and 6-month follow-up, respectively.

Despite having been informed that their risk was not elevated, a large proportion of these women wished to have access to services and particularly breast examination (mentioned by eight (36%) at the 4-week follow-up and 15 (68%) at the 6-month follow-up) and mammography (mentioned by 14 (64%) at the 4-week and 15 (68%) at the 6-month follow-up). At the 4-week follow-up, only five women (23%) stated that they intended to seek further advice (for a variety of reasons) and by the 6-month follow-up, three (14%) had actually sought further advice.

### Relative cost of operating novel and standard service clinics

#### Relative levels of staff costs in the two service models

Based on the duration of appointments, the time taken by staff to carry out related duties, staff travel times, patient attendance rates and the assumptions detailed in the methods section, the novel service staffing and travel costs were approximately 30% lower than those for the standard service.

#### Relative levels of patient costs in the two service models

Questionnaires on time and costs associated with clinic attendance were completed by 164 patients attending the regional clinic and by 104 patients attending a community clinic. The median travel cost for attendance at the regional clinic was £1 (interquartile range, £1–£2.88) and for attendance at a community clinic was £1.00 (interquartile range, £1.00–£1.00). Travel costs were lower for women travelling to a community clinic (*P*<0.001, Mann–Whitney test). The travel time of women attending a community clinic was also less, with 15% of women taking over 30 min to reach the clinic compared to 50% of women attending the regional clinic (*P*<0.001, *χ*^2^ test). In addition, only 2% of women attending a community clinic reported having to arrange care for their children compared to 12% of women attending the regional clinics.

## DISCUSSION

There is widespread recognition of the need to integrate primary- and secondary/tertiary-care services, but the most appropriate model of service delivery remains to be defined (Campbell *et al*, 1995, Donnai *et al*, 2000). This study reports on a cluster randomised trial of a novel model of service delivery and presents patients’ expectations of cancer genetic services and a comparison of patients’ satisfaction with two service models. Patient satisfaction is both an objective and outcome of care, and is therefore an important dimension of any consideration of the best configuration of patient services. In addition, satisfied patients are more likely to comply with advice given, which is an important aspect of any service in which patient information and advice comprises an important element of the intervention (Baker, 1991).

### Expectations of cancer genetic services

About 80% of women stated that they wanted comprehensive information about the implications of their family history of cancer. The items about which women were most concerned to get information or receive services were those connected with their own risk and its possible reduction and early detection of breast cancer. Over 60% of women wanted a breast examination/mammography to have reassurance that they did not have breast cancer and regarded access to breast screening by mammogram and regular check-ups as very important. This underlines the demand for a multidisciplinary service providing both clinical genetic and surgical services, as noted by others (Brain *et al*, 2000). A recognition that increased cancer worry leads to a greater expressed need for information and for reassurance from follow-up checks is also important to guide clinical practice.

### Assessment of cancer genetic clinics

#### Patient satisfaction with services received

Levels of satisfaction with information given, staff attitudes and length of consultation were high. There were no significant differences between the trial groups. The lowest levels of satisfaction were found in those women with levels of cancer risk that were not significantly above population levels, and who were discharged with reassurance only. This reinforces the interpretation that many women seek a clinical examination to allay fears of current cancer (and possibly to have access to future screening such as mammography). It is also consistent with the previous finding that genetic counselling has less impact on general levels of patient satisfaction than other medical procedures, since it rarely ‘suggests treatment or eliminates uncertainties’ (Shiloh *et al*, 1990).

At the 4-week follow-up, 14% of the community clinic (novel service) group and 25% of the regional clinic (standard service) group stated that they intended to seek further advice, although the reasons for this were not primarily due to dissatisfaction with the service they received. The difference between the two low-risk and the two moderate/high-risk groups of women in the trial were not statistically significant. At the 6-month follow-up, only three women had actually attended another clinic for advice. Thus, provision of a community service staffed by nurses did not lead to an increase in the rate of care seeking after the consultation.

#### Clinic preferences

GPs and patients expressed no clear preference for either model of service. Women who had attended a clinic consultation were approximately equally divided between expressing preference for a regional clinic, a community clinic and having no preference. One reason for this may be that many women are working and so may not find it any easier to get to a clinic near their home than to the regional clinic. Similarly, about half of the women had no strong preference when asked for their choice of clinic personnel. Among those who expressed a preference, the combination of being seen by a genetic nurse and consultant breast surgeon was the most popular.

Consultation times were greater when women were seen by a nurse at a community clinic (novel service). This is largely due to the time taken to document the woman's family history, but may also be because women feel more relaxed talking to a nurse or feel reluctant to take up the doctor's time. However, despite the shorter consultation times at the regional clinics, most women were highly satisfied with the duration of all consultations.

### Management of women with a family history of breast cancer, but who do not have an increased risk

In all, 36% of women included in the study were not significantly above population levels of cancer risk. These women were less satisfied with the service received than women with a higher cancer risk. Most of these women were satisfied with the consultation. However, the novel service group was less satisfied than other groups of women with the amount and quality of information given. A relationship between patient satisfaction and rating of comprehension of the information received has been reported (Kincey *et al*, 1975), and failure to reassure has been linked to a failure to provide explanations at women's level of understanding. (Grande *et al*, 2002) It is possible, therefore, that the lower satisfaction reflects explanation and reassurance that is not targeted at the major concerns of these women which are a perceived need for examination for current (and future) cancer rather than principally a need for information about genetic risk. There is a need to tailor the explanation/reassurance by health staff to the background understanding and concerns of these women in order to improve services for these women.

More than a third of low-risk women who attended the community but not the regional clinic stated that they wished access to other services (most often mammography, breast examination, regular check-ups and screening for other cancers) at the 4-week follow-up, although this fell to 22% by the time of the 6-month follow-up. Thus, although most low-risk women were satisfied with being seen by a nurse at a community clinic, many still preferred to have the choice of accessing other services, even after being reassured that their risk is low.

Although most of the low-risk women, who received a letter of reassurance and advice but not a clinic appointment, found the letter quite or very helpful, about a third found it, at most, only a little helpful. A similar percentage said there were other items about which they would have liked information. In all, 50% stated that they wanted a check that they did not have current cancer, 64% that they wanted mammography and 77% that they wanted regular check-ups. At the four-week follow-up, 23% of this group said they intended to seek further advice and at the 6-month follow-up, 14% had actually done so.

### Relative costs associated with the two service models

Since GPs (Campbell *et al*, 2003) and patients expressed no clear preference for any specific service location or staffing configuration, cost is likely to be a major determinant of the nature of these services in the near future. A preliminary comparison of staff time and travel costs in the two trial groups revealed that the novel (community) service was associated with approximately 30% lower staff costs with the assumptions given above. The staff costs of the novel service could be further reduced if the medium/high-risk women were referred to a specialist nurse for breast examination/mammography and did not have a second genetic counselling consultation (since new issues were rarely raised for discussion at this second appointment).

The costs of the standard service could be reduced if the moderate/high-risk patients were assessed at the regional clinic by nurse practitioners who were dually trained in genetics and oncology rather than by a medical consultant or associate specialist staff. This would reduce standard service staff costs to similar levels to the novel service.

It has been previously shown that being seen by nurses trained in breast care (including performing breast examinations for cancer) was acceptable to women and to GPs (Garvican *et al*, 1998). However, any new service model would first require to be evaluated with respect to patient outcomes and patient and staff satisfaction.

Costs to patients in terms of time and money were greater for attendance at the regional centre. This is consistent with the evaluations of other specialist outreach services (Bowling *et al*, 1997). However, since the low-risk patients, who were not offered an appointment at the regional centre, were the least satisfied it would appear that these costs were not a major factor influencing their preference for a particular service.

The potential to decrease nurse/patient contact time could be explored since shorter consultation times (at regional clinics) were not associated with lower levels of patient satisfaction or poorer clinical outcomes. Providing women with written and/or video information about the process and content of genetic counselling prior to their clinic attendance may be one way to achieve this and may in itself contribute to higher levels of patient satisfaction (Austoker and Ong 1994; Hallowell *et al*, 1997; Cull *et al*, 1998).
